# Carcinogenic and non-carcinogenic health risk assessment of heavy metals in drinking water of Khorramabad, Iran

**DOI:** 10.1016/j.mex.2019.07.017

**Published:** 2019-07-19

**Authors:** Ali Akbar Mohammadi, Ahmad Zarei, Saba Majidi, Afshin Ghaderpoury, Yalda Hashempour, Mohammad Hossein Saghi, Abdolazim Alinejad, Mahmood Yousefi, Nasrin Hosseingholizadeh, Mansour Ghaderpoori

**Affiliations:** a Department of Environmental Health Engineering, Neyshabur University of Medical Sciences, Neyshabur, Iran; b Department of Health, School of Public Health, Social Determinant of Health Research Center, Gonabad University of Medical Sciences, Gonabad, Iran; c Department of Environmental Health Engineering, Health Center, Qazvin University of Medical Sciences, Qazvin, Iran; d Student Research Committee School of Public Health, Shahid Beheshti University of Medical Sciences, Tehran, Iran; e Department of Environmental Health Engineering, School of Health, Mazandaran University of Medical Sciences, Sari, Iran; f Department of Environmental Health Engineering, School of Public Health, Sabzevar University of Medical Sciences, Sabzevar, Iran; g Department of Public Health, Fasa University of Medical Sciences, Fasa, Iran; h Department of Environmental Health Engineering, School of Public Health, Tehran University of Medical Sciences, Tehran, Iran; i PhD. Student of Health Education and Promotion, Department of Health Education and Promotion, School of Public Health, Tehran University of Medical Sciences, Tehran, Iran; j Department of Environmental Health Engineering, School of Health and Nutrition, Lorestan University of Medical Sciences, Khorramabad, Iran; k Nutrition Health Research Center, Lorestan University of Medical Sciences, Khorramabad, Iran

**Keywords:** Application of carcinogenic and non-carcinogenic health risk assessment of heavy metals in drinking water, Heavy metals, Non-carcinogenic risk, Carcinogenic risk, Ingestion, Dermal contact

## Abstract

The continuous urbanization and industrialization in many parts of the world and Iran has led to high levels of heavy metal contamination in the soil and then on the surface and groundwater. In this study, the concentrations of 8 heavy metals were determined in forty water samples along distribution drinking water of Khorramabad, Iran. The ranges of heavy metals in this study were lower than EPA and WHO drinking water recommendations and guidelines and so were acceptable. The mean values of CDI_total_ of heavy metals concentrations in adults were found in the order of Zn > Ba > Pb > Ni > Cr > Cu > Cd > Mo. The health-risk estimation indicated that total hazard quotient (HQ_ing_ + HQ_derm_) and hazard index values were below the acceptable limit, representing no non-carcinogenic risk to the residents via oral intake and dermal adsorption of water. Moreover, the results of total risk via ingestion and dermal contact showed that the ingestion was the predominant pathway. This study also presents that the carcinogenic risk for Pb, Cr, Cd and Ni were observed higher than the acceptable limit (1 × 10^−6^). The present study will be quite helpful for both inhabitants in taking protective measures and government officials in reducing heavy metals contamination of urban drinking water.

• The data analyzed in this study show a clear situation regarding the quality of drinking water in Khorramabad.
• The results of this study can be used to improve and develop the quality of drinking water that directly affects the health of consumers.
• The present study will be quite helpful for both inhabitants in taking protective measures and government officials in reducing heavy metals contamination of urban drinking water

## Graphical abstract

**Figure:**
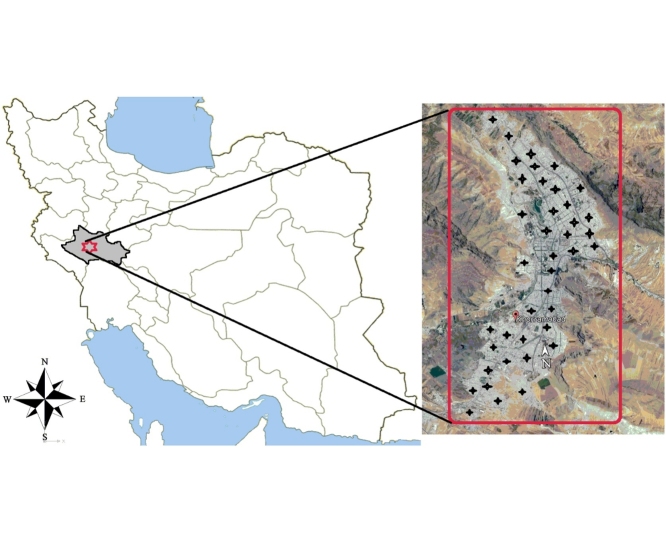

**Specifications Table**

**Table:** 

Subject area:	Environmental Science
More specific subject area:	Drinking water monitoring and quality
Method name:	Application of carcinogenic and non-carcinogenic health risk assessment of heavy metals in drinking water
Name and reference of the original method:	Concentration and ecological risk of heavy metal in street dust of Eslamshahr, Iran. *Human and Ecological Risk Assessment: An International Journal*, 1-10
Resource availability:	The data are available with this article.

## Method details

Supply of healthy drinking water is necessary to human life, and safe drinking water should not cause a remarkable risk to human health. The increasing trend of water shortage has various negative impacts on economic development, human livelihoods, and environmental quality around the world [[1], [2], [3]]. Numerous contaminants, including heavy metals, organic and inorganic compounds, etc. may contaminate water. Among harmful and persistent contaminants found in water, a special emphasis is given to heavy metals [4,5]. Rapid economic development and industrialization in many parts of the world and Iran has led to high levels of heavy metal contamination in the soil and then in the surface and groundwater [[6], [7], [8], [9], [10]]. The heavy metals are released into the water naturally or via human activities [11,12]. Many heavy metals are the natural elements of the earth’s crust. Weathering and decomposition of metal rock and ores can transfer heavy metals in groundwater and have led to human exposure for the entire history of mankind [5,13,14]. The levels of metals vary significantly from the soil of one region to another [15]. Anthropogenic activities considerably affect the availability of heavy metals in the ecosystems. Heavy metals may be released into water the in large quantities via vehicle exhaust, poor waste disposal, fossil fuel combustion, fertilizer and pesticide application, untreated wastewater irrigation, and atmospheric precipitation from various human activities including mining, smelting operation, agriculture, etc. which can influence human health by affecting on vegetation, food chain and water quality [16]. Once released into the drinking water, heavy metals can be taken into the human body through several pathways such as direct ingestion, dermal contact, inhalation, through mouth and nose [17]. Heavy metals in water can cause extensive damage to the ecological environment and consequently human health due to their unique characteristics such as toxicity, poor biodegradability and bioaccumulation [[18], [19], [20], [21]]. Some heavy metals are detrimental for metabolisms in the human body, serving as both structural and catalytic constituents of proteins and enzymes, but can have adverse effects when the levels were greater than international guidelines [22]. During prolonged exposure, heavy metals can accumulate in target tissues such as brain, liver, bones, and kidneys in the human body resulting in serious health hazards, depending on the element and its chemical form [23]. Health risk assessment of heavy metals is usually performed to estimate the total exposure to heavy metals among the residents in a particular area. Risk assessment of contaminants in humans is based on a mechanistic assumption that such chemicals may either be carcinogenic or non-carcinogenic [24]. Generally, ingestion and dermal absorption are the major pathways of exposure in water environment [25,26]. In order to assess water quality in an area effectively, it is crucial to find possible human health impacts of contaminants in drinking water. The traditional technique for estimating health impacts is to directly compare the analyzed levels with guideline limits, but it is not adequately valid to provide comprehensive hazard levels and find contaminants of the most important [27]. Health risk assessment is an essential method for evaluating the possible health effects in water environments caused by numerous contaminants [28,29]. This method has been extensively utilized by many researchers in literature for the estimation of the adverse health effects possible from exposure to contaminated water [30,31]. Although ingestion is the predominant pathway of exposure to contaminants in drinking water, inhalation and dermal absorption should also be considered [24]. Most health risk estimations associated with human exposure to contaminants in soil, water, and air are based on the exposure methods presented by the USEPA [13]. With the increasing trend of population, economy, and industry growth in Iran, the study is required to determine the impacts of development on the surface and groundwater, before any preventive measures can be considered in the land-use systems and watersheds to decrease the contamination levels of heavy metals. The main objectives of the present research were to determine levels of eight heavy metals including Lead (Pb), Chromium (Cr), Cadmium (Cd), Molybdenum (Mo), Zinc (Zn), Copper (Cu), Barium (Ba), and Nickel (Ni) in the drinking water of Khorramabad city and estimate health risks of non-carcinogenic (Pb, Cr, Cd, Mo, Zn, Cu, Ba, and Ni) and carcinogenic (Pb, Cr, Cd, and Ni) metals with respect to daily drinking of groundwater and dermal pathways for general adults in the community. The results of our research may provide some insight into heavy metal contamination in water and are useful for inhabitants in formulating protective procedures and health professionals in reducing heavy metal contamination of water environment, and also serve as a basis for comparison to other areas both in Iran and worldwide.

## Materials and methods

### Study area description

**Fig. 1:**
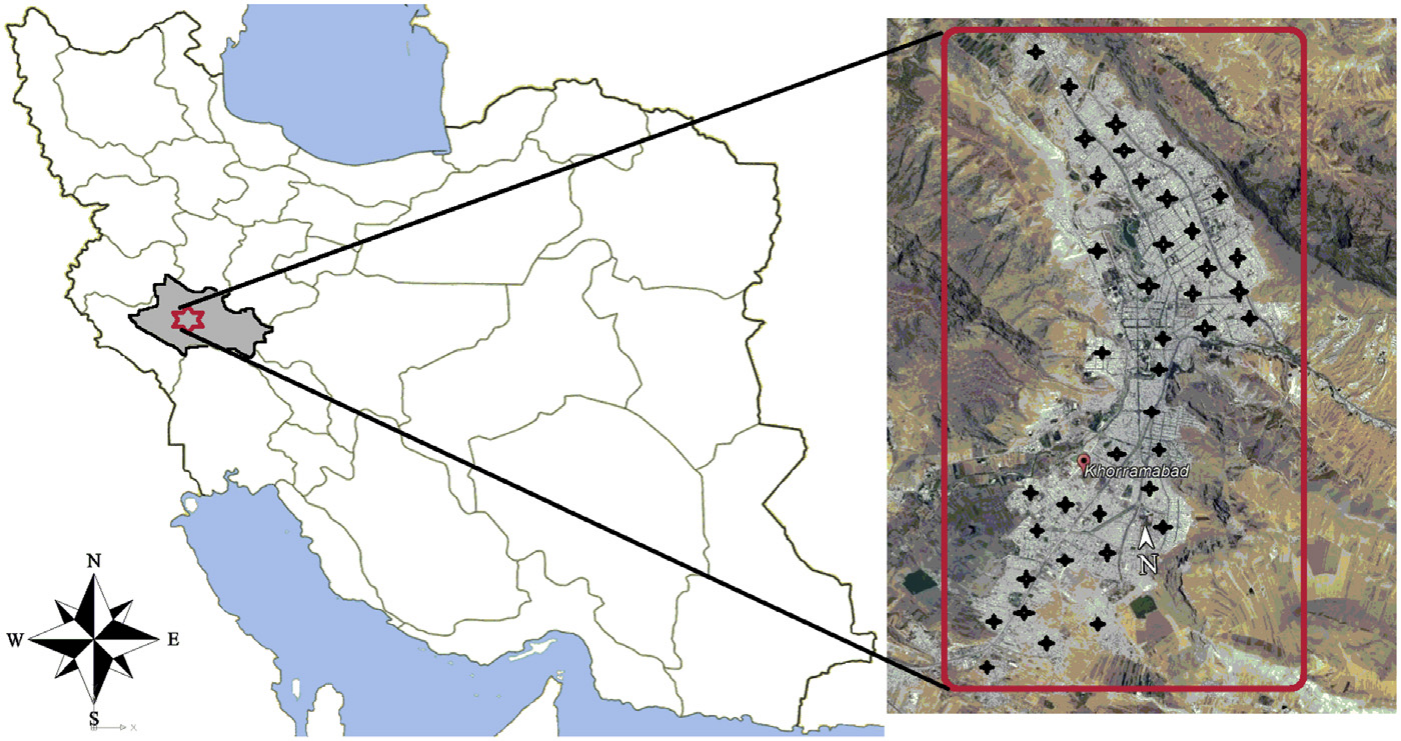
Location map of sampling sites in the distribution network.

The geographic coordinates of the study area are 33°29′16″N 48°21′21″E in DMS (Degrees Minutes Seconds), located in the Khorramabad city, Lorestan Province in the west of Iran. Khorramabad is situated in the Zagros Mountains with a warm and temperate climate. Natural springs are the main sources of water supply in this city. At the 2016 census, its population was 373,416. Average annual rainfall in this region is 488 mm. This city stands at an elevation of approximately 1147 m above sea level [32]. The location map of the study area is depicted in Fig. 1.

### Materials and sampling

Analytical grade HNO_3_ purchased from Merck Company was used in this work. Deionized water was utilized for solution preparation and also for dilution objectives. All glassware was washed and dried in an oven at 105 °C. Sampling bottles were cleaned by rinsing in a metal-free soap and then by soaking in 10% HNO_3_ before sample taking. Finally, the bottles were washed with deionized water. Totally, forty water samples from 40 different sites along the distribution network were collected during 2017 in order to measure the levels of potentially toxic heavy metals such as Pb, Cr, Cd, Mo, Zn, Cu, Ba, and Ni in drinking water of Khorramabad city. These samples were then transported to the laboratory and stored at 4 °C until analysis.

### Sample analysis

The collected samples were analyzed for eight heavy metals including Pb, Cr, Cd, Mo, Zn, Cu, Ba, and Ni using standard methods for the examination of water and wastewater [33]. Concentrations of the heavy metals in all samples were measured using an inductively coupled plasma mass spectrometry (ICP-MS). The limit of detection (LOD) of individual metal was in the range 0.5–5 ng/L for water samples.

### Health risk assessment

#### Non-carcinogenic analysis

##### Risks of individual heavy metals

Risk assessment is defined as the methods of evaluating the probability of occurrence of any given probable amount of the harmful health impacts over a determined time period [34]. The health risk assessment of each contaminant is normally based on the estimation of the risk level and is classified as carcinogenic or non-carcinogenic health hazards [35]. To estimate the heavy metal contamination and potential carcinogenic and non-cancer health risk caused via ingestion and dermal absorption of heavy metals in the water of the distribution network of Khorramabad city, Hazard Quotients (HQ), Hazard Index (HI), and the Incremental Lifetime Cancer Risk (ILCR) were used. The studied group in this study was adults.

Eq. 1 and Eq. 2 taken from the Environmental Protection Agency (USEPA) were applied to determine the Chronic Daily Intake (CDI) via ingestion and dermal absorption routes, respectively [25,36].

(1) $${\text{C}\text{D}\text{I}\;}_{\text{i}\text{n}\text{g}\text{e}\text{s}\text{t}\text{i}\text{o}\text{n}}=\frac{{\text{C}}_{\text{w}}.\text{D}\text{I}.\text{A}\text{B}\text{S}.\text{E}\text{F}.\text{E}\text{P}}{\text{B}\text{W}.\text{A}\text{T}}$$

(2) $${\text{C}\text{D}\text{I}}_{\;\text{d}\text{e}\text{r}\text{m}\text{a}\text{l}}=\frac{{\text{C}}_{\text{w}}.\text{S}\text{A}.{\text{K}}_{\text{p}}.\;\text{A}\text{B}\text{S}.\text{E}\text{T}.\text{E}\text{F}.\text{E}\text{P}.\text{C}\text{F}}{\text{B}\text{W}.\text{A}\text{T}}$$

Where, ${\text{C}}_{\text{w}}$ (in mg/L) is the concentration of heavy metals in water, SA (${\text{i}\text{n}\;\text{c}\text{m}}^{2}$) is the skin area available for contact, ${\text{K}}_{\text{p}}$ (in cm/hour) is the permeability coefficient, ABS (unitless) is the dermal absorption factor, DI (in L/day) is the daily average intake of water in the area, ET (in h/event) is the exposure time, EF (in days/year) represents the annual exposure frequency, ED (in years) is exposure period, CF (in $\text{L}/{\text{c}\text{m}}^{3}$) is the unit conversion factor, BW (in Kg/person) is body weight, and AT (in days) is the average time. The input assumptions and their values for computing the chronic daily intake through oral ingestion and dermal absorption are summarized in Table 1.

**Table 1:** Parameters and input assumptions for exposure assessment of metals through ingestion and dermal pathways.

Parameter	Unit	Values	
		Ingestion	Dermal adsorption
Heavy metal concentrations (${\text{C}}_{\text{w}}$)	μg/L	–	–
Daily average intake (DI)	L/day	2.2	–
Skin-surface area (SA)	Cm^2^	–	18000
Permeability coefficient (Kp)	Cm/hour	–	Pb, Cu, Ba 0.001, Cr, 0.002, Zn 0.006, Ni 0.0002
Exposure time (ET)	Hour/event	–	0.58
Exposure frequency (EF)	Day/years	365	350
Exposure duration (EP)	Year	70	30
Conversion factor (CF)	L/cm^3^		0.001
Body weight (BW)	Kg	70	70
ABS	All	0.001	0.001
Average time (AT)	Days	25550	25550

The HQ for each heavy metal was estimated using the ratio of computed mean daily intake (ADI, mg/kg/day) of a metal ingested with contaminated water to the reference oral dose (RfD) through oral ingestion and dermal absorption for the residents. The sum of all HQs gives an estimation of total potential health risks or HI. The calculation of the HI caused by water is presented as (Eq. 3):

(3) $$\text{H}\text{Q}=\frac{\text{C}\text{D}\text{I}}{\text{R}\text{f}\text{D}}$$

Where, CDI and RfD are expressed in mg/kg-day.

The values of the RfD and cancer slope factor for different metals are listed in Table 2.

**Table 2:** Reference dose (RfD) and cancer slope factor (CSF) for different metals.

Element	Rdf_oral_	Rdf_dermal_	CSF (kg/day/mg)
Pb	1.4	0.42	8.5
Cr	3	0.015	41
Cd	0.5	0.005	6.1
Mo	5	1.9	
Zn	300	60	
Cu	40	12	
Ba	70	14	
Ni	20	5.4	0.84

##### Hazard Index (HI) for multiple heavy metals

To estimate the total potential non-carcinogenic health impacts caused by exposure to a mixture of heavy metals in water, the HI for several heavy metals was computed according to the EPA guidelines for health risk assessment [37,38] using following Eq. 4:

(4) $$\text{H}\text{I}=\sum_{\text{k}=1}^{\text{n}}\text{H}\text{Q}={\text{H}\text{Q}}_{\text{P}\text{b}}+{\text{H}\text{Q}}_{\text{C}\text{r}\;}+{\text{H}\text{Q}}_{\text{C}\text{d}\;}+{\text{H}\text{Q}}_{\text{M}\text{o}}+{\text{H}\text{Q}}_{\text{Z}\text{n}}+{\text{H}\text{Q}}_{\text{C}\text{u}}+{\text{H}\text{Q}}_{\text{B}\text{a}\;}+{\text{H}\text{Q}}_{\text{N}\text{i}}$$

The computed HI is compared to standard values: there is the possibility that non-carcinogenic impacts may occur in the residents when HI > 1, while the exposed person is unexpected to experience evident harmful health impacts when HI < 1 [39].

#### Carcinogenic analysis

The probable cancer risks due to exposure to a specified dose of heavy metal in drinking water can be computed using the ILCR [40]. The ILCR is defined as the incremental probability of a person developing any type of cancer over a lifetime as a result of twenty-four hours per day exposure to a given daily amount of a carcinogenic element for seventy years [41]. The following equation (Eq. 5) was commonly used for the calculation of the lifetime cancer risk:

(5) ILCR=CDI .CSF

Where, CSF is the cancer slope factor and is defined as the risk generated by a lifetime average amount of one mg/kg/day of carcinogen chemical and is contaminant specific.

The permissible limits are considered to be 10^−6^ and <10^−4^ for a single carcinogenic element and multi-element carcinogens [42].

## Results

The minimum, mean, and maximum levels of heavy metals (Pb, Cr, Cd, Mo, Zn, Cu, Ba, and Ni) present in water samples in the distribution network of Khorramabad city are presented in Table 3. The minimum, mean, and maximum levels of CDI, as well as total CDI for adults through ingestion and dermal contact pathways in the study area, are given in Table 4. The minimum, mean, and maximum levels of HQ, as well as total HQ for adults through ingestion and dermal contact pathways, are presented in Table 5. The carcinogenic risk assessment for adults is given in Table 6.

**Table 3:** Heavy metal concentrations in the water distribution network of the study area.

		Heavy metal concentrations (μg/L)		Drinking groundwater standard (μg/L)	
Metal	Mean	Minimum	Maximum	USEPA (2012)	WHO (2011)
Pb	3.2	0.35	8.27	15	10
Cr	5.08	0.39	10.76	100	50
Cd	0.43	0.00	1.49	5	3
Mo	0.51	0.07	1.05	Not mentioned	70
Zn	47.01	7.41	104.77	5000	Not mentioned
Cu	6.79	0.10	39.31	1300	2000
Ba	81.13	27.60	173.15	2000	700
Ni	3.47	0.06	19.45	Not mentioned	70

**Table 4:** Chronic daily intake (CDI) for heavy metals through different pathways.

	CDI_ing_			CDI_der_			CDI_total_		
	mean	min	max	mean	min	max	mean	min	max
**Pb**	1.00E-04	1.10E-05	2.60E-04	1.96E-08	2.15E-09	5.07E-08	1.00E-04	1.10E-05	2.60E-04
**Cr**	1.60E-04	1.23E-05	3.38E-04	3.11E-08	2.39E-09	6.59E-08	1.60E-04	1.23E-05	3.38E-04
**Cd**	1.34E-05	3.14E-08	4.67E-05	2.61E-09	6.13E-12	9.11E-09	1.34E-05	3.14E-08	4.67E-05
**Mo**	1.60E-05	2.20E-06	3.30E-05	3.13E-09	4.29E-10	6.44E-09	1.60E-05	2.20E-06	3.30E-05
**Zn**	1.48E-03	2.33E-04	3.29E-03	2.88E-07	4.54E-08	6.42E-07	1.48E-03	2.33E-04	3.29E-03
**Cu**	2.13E-04	3.14E-06	1.24E-03	4.16E-08	6.13E-10	2.41E-07	2.13E-04	3.14E-06	1.24E-03
**Ba**	2.55E-03	8.67E-04	5.44E-03	4.97E-07	1.69E-07	1.06E-06	2.55E-03	8.68E-04	5.44E-03
**Ni**	1.09E-04	1.89E-06	6.11E-04	2.13E-08	3.68E-10	1.19E-07	1.09E-04	1.89E-06	6.11E-04

**Table 5:** Mean, minimum, and maximum values of non-carcinogenic human health risks posed by heavy metals in water of study area via different pathways.

H	HQ_ing_			HQ_der_			HQ_total_		
Metal	mean	min	max	mean	min	max	mean	min	max
Pb	7.17E-05	7.86E-06	1.86E-04	4.66E-08	5.11E-09	1.21E-07	7.18E-05	7.86E-06	1.86E-04
Cr	1.14E-04	8.76E-06	2.42E-04	7.41E-08	5.69E-09	1.57E-07	1.14E-04	8.76E-06	2.42E-04
Cd	9.58E-06	2.24E-08	3.34E-05	6.23E-09	1.46E-11	2.17E-08	9.58E-06	2.25E-08	3.34E-05
Mo	1.14E-05	1.57E-06	2.36E-05	7.44E-09	1.02E-09	1.53E-08	1.15E-05	1.57E-06	2.36E-05
Zn	1.06E-03	1.66E-04	2.35E-03	6.86E-07	1.08E-07	1.53E-06	1.06E-03	1.66E-04	2.35E-03
Cu	1.52E-04	2.24E-06	8.82E-04	9.90E-08	1.46E-09	5.74E-07	1.52E-04	2.25E-06	8.83E-04
Ba	1.82E-03	6.20E-04	3.89E-03	1.18E-06	4.03E-07	2.53E-06	1.82E-03	6.20E-04	3.89E-03
Ni	7.78E-05	1.35E-06	4.37E-04	5.06E-08	8.76E-10	2.84E-07	7.79E-05	1.35E-06	4.37E-04
**HI**	3.31E-03	8.08E-04	8.04E-03	2.15E-06	5.25E-07	5.23E-06	3.32E-03	8.08E-04	8.05E-03

**Table 6:** The incremental lifetime cancer risk (ILCR) values of carcinogenic human health risks via total exposure (ingestion and dermal contact) to the drinking water of the study area for adults.

ILCR			Metal
Max	Min	Mean	
2.21 E-03	9.35E-05	8.54E-04	Pb
1.39E-02	5.03E-04	6.54E-03	Cr
2.85E-04	1.92E-07	8.18E-05	Cd
5.14E-04	1.58E-06	9.16E-05	Ni
1.47 E-02	5.05 E-04	7.57 E-03	∑ILCR

## Discussion

The heavy metal contamination in water distribution network can increase human health risks through various exposure routes. In the present work, non-carcinogenic and carcinogenic health risks caused by oral ingestion and dermal contact were explored. Based on Table 3, a wide variation in mean values of heavy metals was seen in the water where the maximum metal concentration was for Ba with a mean of 81.13 mg/L and the minimum metal concentration was for Cd with a mean concentration of 0.43 mg/L, respectively. The order of the toxicity heavy metals according to mean concentrations measured in drinking water of the studied area was: Ba > Zn > Cu > Cr > Ni > Pb > Mo > Cd.

### Non-carcinogenic analysis

Human health risk assessment comprises the determination of the nature and magnitude of adverse health effects in humans who may be exposed to toxic substances in a contaminated environment. In the present work, exposure and risk assessments were carried out based on the USEPA methodology. Human exposure to heavy metals principally occurs via pathways of drinking water, food, inhaled aerosol particles and dust [43]. The degree of toxicity of heavy metals to human health is directly related to their daily intake. However, ingestion via drinking water and dermal adsorption was considered in this study. The first step in the non-carcinogenic analysis is the calculation of chronic daily intake (CDI) values. As given in the Table 4, the mean levels of total CDI (CDI_total_) in mg/kg-day are 1.00E-04 for Pb, 11.60E-04 for Cr, 1.34E-05 for Cd, 1.60E-05 for Mo, 1.48E-03 for Zn, 2.13E-04 for Cu, 2.55E-03 for Ba, and 1.09E-04 for Ni. Therefore, the mean values of CDI_total_ of heavy metals concentrations for adults were found in the order of Zn > Ba > Pb > Ni > Cr > Cu > Cd > Mo.

As seen in Table 5, all the studied heavy metals had total HQs below 1. Accordingly, the health risk estimation of Pb, Cr, Cd, Mo, Zn, Cu, Ba, and Ni revealed the mean HQs suggesting an acceptable level of non-carcinogenic harmful health risk in all samples taken from Khorammabed’s water distribution network. From the computation of total HQs, it can be concluded that the contribution of the eight metals to the non-carcinogenic health risk was in the order of Zn > Ba > Cr > Cu > Mo > Pb > Ni > Cd.

Moreover, to estimate the total potential non-carcinogenic impacts induced by more than one metal, the HQ computed for each metal is summed and expressed as a Hazard Index (HI) [44]. The mean values of HI through ingestion and dermal adsorption as wells as total HI were obtained to be 3.31E-03, 2.15E-06, and 3.32E-03, respectively. It shows neglectable non-carcinogenic risk to residents’ health as the value of HI is below 1. The values of HI for heavy metals of inhabitants in the study area are summarized in Table 5.

### Carcinogenic risk analysis

Heavy metals (Pb, Cr (VI), Cd, and Ni) can potentially enhance the risk of cancer in humans [45,46]. Long term exposure to low amounts of toxic metals could, therefore, result in many types of cancers. Using Pb, Cr (VI), Cd, and Ni as carcinogens, the total exposure of the residents were assessed based on the mean CDI values given in Table 4. The carcinogenic risk assessment for adults is given in Table 6. The values of cancer slope factor (CSF) for different metals used for carcinogenic risk assessment are listed in Table 2.

For one heavy metal, an ILCR less than 1 × 10^−6^ is considered as insignificant and the cancer risk can be neglected; while an ILCR above 1 × 10^-4^ is considered as harmful and the cancer risk is troublesome. For the total of all heavy metals through all exposure routes, the acceptable level is 1 × 10^-5^ [[46], [47], [48]]. Among all the studied heavy metals, chromium has the highest chance of cancer risks (mean ILCR 6.54 × 10^-3^) and nickel has the lowest chance of cancer risk (mean ILCR 9.16 × 10^-5^). The results of this research present that there was a cancer risk from the contaminants to residents through the cumulative ingestion and dermal contact routes in the drinking water of the region.

## Conclusions

This study was conducted to evaluate the health risks of exposure to heavy metals along with the water distribution network of Khorramabad city in Iran. Risk assessment relevant for the present study comprises computations of carcinogenic and non-carcinogenic risk of water through ingestion and dermal contact pathways. The maximum and minimum concentrations of heavy metals measured were related to Ba (81.31 mg/L) and Cd (0.43 mg/L), respectively. The order of the heavy metals toxicity according to mean concentrations measured in drinking water of the studied area was: Ba > Zn > Cu > Cr > Ni > Pb > Mo > Cd. Themean values of CDI_total_ of heavy metals concentrations in adults were found in the order of Zn > Ba > Pb > Ni > Cr > Cu > Cd > Mo. The HQs for those routes of this work decline in the following order: ingestion > dermal adsorption, meaning that ingestion is the dominant pathway of exposure to every receptor. The mean values of HI through ingestion and dermal adsorption as wells as total HI were obtained to be 3.31E-03, 2.15E-06, and 3.32E-03, respectively. Among all the studied heavy metals, chromium has the highest chance of cancer risks (mean ILCR 6.54 × 10^−3^) and nickel has the lowest chance of cancer risk (mean ILCR 9.16 × 10^-5^). The present study will be quite helpful for both inhabitants in taking protective measures and government officials in reducing heavy metals contamination of urban drinking water.

## Declaration of Competing Interest

The authors of this article declare that they have no conflict of interests.
